# Benchmarking gambling screens to health-state utility: the PGSI and the SGHS estimate similar levels of population gambling-harm

**DOI:** 10.1186/s12889-022-13243-4

**Published:** 2022-04-27

**Authors:** Matthew Browne, Alex M. T. Russell, Stephen Begg, Matthew J. Rockloff, En Li, Vijay Rawat, Nerilee Hing

**Affiliations:** 1 School of Medical, Health & Applied Sciences, Central Queensland University, Bundaberg B8 G.47 University Dr, Branyan, QLD 4670 Australia; 2 La Trobe Rural Health School, College of Science, Health and Engineering, La Trobe University, Melbourne, Australia

**Keywords:** Gambling harms, Gambling problems, Health utility, SF-6D, Problem gambling severity index, Short gambling harms screen

## Abstract

**Background:**

Both the Problem Gambling Severity Index (PGSI) and the Short Gambling Harms Screen (SGHS) purport to identify individuals harmed by gambling. However, there is dispute as to how much individuals are harmed, conditional on their scores from these instruments. We used an experienced utility framework to estimate the magnitude of implied impacts on health and wellbeing.

**Methods:**

We measured health utility using the Short Form Six-Dimension (SF-6D), and used this as a benchmark. All 2603 cases were propensity score weighted, to balance the affected group (i.e., SGHS 1+ or PGSI 1+ vs 0) with a reference group of gamblers with respect to risk factors for gambling harm. Weighted regression models estimated decrements to health utility scores attributable to gambling, whilst controlling for key comorbidities.

**Results:**

We found significant attributable decrements to health utility for all non-zero SGHS scores, as well as moderate-risk and problem gamblers, but not for PGSI low-risk gamblers. Applying these coefficients to population data, we find a similar total burden for both instruments, although the SGHS more specifically identified the subpopulation of harmed individuals. For both screens, outcomes on the SF-6D implies that about two-thirds of the ‘burden of harm’ is attributable to gamblers outside of the most severe categories.

**Conclusions:**

Gambling screens have hitherto provided nominal category membership, it has been unclear whether moderate or ‘at-risk’ scores imply meaningful impact, and accordingly, population surveys have typically focused on problem gambling prevalence. These results quantify the health utility decrement for each category, allowing for tracking of the aggregate population impact based on all affected gamblers.

**Supplementary Information:**

The online version contains supplementary material available at 10.1186/s12889-022-13243-4.

## Background

How bad is it to have gambling problems or to experience gambling-related harm? Much gambling research rests on the use of population screens to measure these phenomena [1]. These screens yield categories, such as low-risk, moderate-risk or problem gambler (LR, MR, PG) on the Problem Gambling Severity Index (PGSI) [2], or scores: 0-10, in the case of the Short Gambling Harms Screen (SGHS) [3]. However, there is controversy as to what these measures indicate in terms of negative impact actually experienced by the self-reporting gambler [4–6]. Are LR or MR gamblers genuinely impacted, or are they merely *at-risk* of meaningful impact? Likewise, are low (e.g., 1-2) scores on the SGHS truly indicative of meaningful harm, or might they merely reflect rational opportunity costs [5]? These basic questions have large ramifications, not only for how these screens should be interpreted at an individual level, but also regarding their use in capturing the distribution and extent of impacts from gambling in populations, and the targeting of policy interventions for harm reduction [7–10].

In their summary of the evolution of population assessment of gambling impacts, Browne et al. [1] argue that scales for harm must be assessed with regard to external benchmarks. There has been detailed enumeration of the population prevalence of specific gambling-related harms, such as having sold personal items due to gambling [11–13]. However, these do not, except in a qualitative or implied sense, address the question of how subjectively *bad it is* to experience these consequences. Some limited work has been done to further this goal. Blackman et al. [14] found monotonic decrements in subjective wellbeing across the three PGSI risk categories, relative to non-problem gamblers (NPGs). Similarly, Hilbrecht and Mock [15] found lower levels in several facets of quality of life for LR and MR gamblers relative to NPG on the PGSI. Similarly, the SGHS was validated against the Personal Wellbeing Index (PWI), showing a monotonic and approximately linear correlation, with lower PWI scores associated with higher SGHS scores [3]. Most recently, relatively low scores of 1-2 on the SGHS were shown to be associated with significant higher psychological distress (Kessler) and lower wellbeing (PWI), compared to those who scored 0 [16].

Prior work demonstrates that gambling screens are associated with external measures that imply impacts to global health and wellbeing [14, 15]. However, in some sense, these results simply kick the can further down the road, begging the question of which external metrics are most relevant, and how decrements on these benchmarks should themselves be interpreted. Recently, a case has been made for the central role of *health utility* as the key yardstick for scoring gambling screens [17]. In that paper, an approach was outlined for employing global health utility instruments to assess gambling. We will briefly reprise this rationale, which will be applied in this study.

### Health utility as a benchmark for gambling impact

There is general agreement that impact from gambling is best understood as “*a decrement to the health or wellbeing of an individual…*” [18]. The public health / health economic framework of health utility [19] operationalises this concept – since gambling harm is understood as a decrease to a person’s health-related quality of life. A drop in health and wellbeing is an anti-hedonic outcome that is, by definition, something that an individual would prefer to avoid. Crucially, and unlike other candidate benchmarks, health utility is measured on a metric scale, where a score of 1 corresponds to optimal health, and a score of 0 corresponds to a health state judged to be not worth living, or equivalent to death [20]. Making the democratic assumption that every individual’s utility is equally important, then optimal population health can be effected by maximising the integral over the lifespan and over people.

These attractive theoretical properties justify the central role of health utility across many disciplines concerned with public health. However, estimating or eliciting the typical decrement associated with a condition (e.g, alcohol abuse or problem gambling) is less straight-forward. Protocols such as the Time Trade Off, the Standard Gamble or the Visual Analogue Scale are used to elicit *preference-based utilities*, based on providing raters with some stimuli that describe the experience of the condition. Also known (somewhat confusingly) as direct elicitation paradigms, they rely on the ability of respondents to accurately imagine the experience of the condition, and to judge a relative preference for hypothetical scenarios with- and without the condition. As delineated elsewhere [21–23], there are inherent biases and limitations to these procedures. Nevertheless, when combined with rank-ordering methods between conditions, and accounting for co-morbidities, these methods have been used to great effect to assess the relative contribution of conditions in the Global Burden of Disease framework, and specifically for mental and addictive disorders [24]. Direct elicitation methods have also been applied to assess utility weights for PGSI categories [25–28], finding preference weights for gambling that are similar to those for mild, moderate and severe alcohol misuse. However, there are challenges to preference-based utilities, such that (1) assessors may struggle to imagine the net effect of living with a given degree of gambling problems, (2) there may be framing effects associated with popular conceptions of problem gambling, and (3) the decrement may be anchored to a counterfactual that assumes an ideal state of health and wellbeing (i.e., 1) that is unlikely to be realistic for most respondents (i.e., few people are in a perfect state of mental and physical health).

Dolan and Kahneman [21] provide arguments in favour of *experienced utility* as opposed to decision or preference-based utility to assess the impact of a condition. In this framework, persons with- and without the condition (i.e., some degree of gambling problems or harms) are asked about their own experienced quality of life. After weighting and/or case matching with controls, and controlling for major co-morbidities, the relative difference in self-reported health is attributed to the condition. However, given an observed decrement, this procedure presents its own challenges in attributing causality to the condition. First, the study should approximate an experimental random assignment, such that individuals in both groups have the same *propensity* to experience gambling problems or harms. This is important to reduce confounding, because the risk factors that lead some individuals to have a propensity for gambling problems may also contribute to a lower health status due to other sources of harm. Second, when estimating the direct effect of a gambling-related condition on health utility, known co-morbid conditions that might also affect the outcome should be controlled for [29, 30]. This control is a second way to avoid attributing the impact of co-occurring conditions to the gambling, rather than to co-occurring conditions. Thus, unlike other attempts to estimate health utility impacts from gambling screens [31], this framework includes both propensity and causal modelling components, and requires identification of relevant risk-factors and co-morbid conditions. We again refer the reader to Browne et al. [17] for a more detailed overview and rationale for this framework as applied to gambling-related harm, as well as a review of relevant risk-factors and comorbidities for gambling problems.

### Aims

The present study attempts to implement an indirect elicitation approach to estimate the health utility impacts for any gambling screen. Our objective was to estimate metric (0,1) health utility weights for two common population screens for gambling impact: the SGHS and the PGSI using an experienced utility / propensity score weighting approach. The SGHS measures gambling harm whereas the PGSI measures problem gambling, although these constructs are highly correlated, and both are expected to be related to reductions in wellbeing.

## Method

Our analysis was based on a comparison of health utility scores between unharmed / non-problem gamblers, for the SGHS and PGSI, respectively, who had participated in gambling at least once in the last year (hereafter, the *control* group) and those experiencing some degree of harm or problems (hereafter, the *affected* group). It is important to note that the control group for the SGHS and PGSI analyses were slightly different, as some respondents may have scored 0 on the SGHS and therefore been in the control group for SGHS-based analyses, but scored more than 0 on the PGSI and therefore been in the affected group for PGSI-based analyses. Sampling was stratified with respect to group, age and gender. Cases were propensity weighted based on key risk factors, and regression-estimated coefficients were estimated with control variables for gambling comorbidities. Similar analyses were run using the PGSI and the SGHS to define the reference (score 0) and affected (score 1+) groups. Categorical, linear and non-linear utility functions of 1+ scores were compared.

### Participants

Australian participants aged 18+ were recruited from a commercial panel provider during late 2020 and early 2021 as part of a broader project to study gamblers, non-gamblers, and ‘concerned significant others’. The commercial panel has their network of respondents who have signed up to take part in research opportunities. The panel invited respondents through email and all data was collected online. As compensation, participants received points which could be exchanged for rewards as per the panel’s internal points-accumulation system.

All eligible participants were required to be Australian residents, aged 18 years or above, provide consent to participate in the study, and to have gambled^1^ in the past 12 months. Residents of the state of Victoria were excluded due to COVID lockdown at time of sampling. Using soft-quotas, we attempted to sample approximately equal groups with respect to age (18-29, 30-44, 45+) and gender with respect to control / affected group status. A total of 22,699 started the survey, however 16,061 were screened out for the following reasons: 5848 did not meet the residency or age criteria, 5922 provided incomplete responses, 441 provided poor quality data (such as straight lining through the survey), and 3850 were excluded due to quotas being full. A total of 6638 responses were retained, of which 2603 were gamblers and formed part of the present analysis, with 1193 (45.8%) scoring zero on *both* population screens. Table 1 provides the demographic characteristics for gamblers and figures are presented separately for gamblers who scored zero and 1+ on *each* screen. For the SGHS 1546 gamblers (59%) scored 0 and 1057 (41%) scored 1+, and for the PGSI 1331 (51%) scored 0 and 1272 (49%) scored 1+. The most common forms gambled on included lotteries (82.1% of sample), electronic gaming machines (65.3%), scratch tickets (64.0%), race betting (63.8%), raffle tickets / competitions (62.9%), sports betting (43.6%), and Keno (41.3%). Less than one-third of participants gambled on all other forms (casino table games, informal private betting, prize draws, Bingo, eSports, fantasy sports, and ‘other’).

**Table 1:** Descriptive statistics for the sample of gamblers, by SGHS and PGSI reference and affected groups

	Reference		Affected	
Variable	SGHS 0 n (%)	PGSI 0 n (%)	SGHS 1+ n (%)	PGSI 1+ n (%)
**Total**	**1546 (100)**	**1331 (100)**	**1057 (100)**	**1272 (100)**
*Gender*				
Male	880 (56.9)	758 (56.9)	613 (58.0)	735 (57.8)
Female	665 (43.0)	572 (43.0)	443 (41.9)	536 (42.1)
Other	1 (0.1)	1 (0.1)	1 (0.1)	1 (0.1)
*Mean age* (SD [years])	51.16 (17.48)	52.14 (17.13)	42.15 (16.03)	42.65 (16.48)
*Country of birth*				
Australia	1232 (79.7)	1068 (80.2)	845 (79.9)	1009 (79.3)
Other	314 (20.3)	263 (19.8)	212 (20.1)	263 (20.7)
*Main language spoken at home*				
English	1497 (96.8)	1296 (97.4)	991 (93.8)	1192 (93.7)
Other	49 (3.2)	35 (2.6)	66 (6.2)	80 (6.3)
*Aboriginal or Torres Strait Islander origin*				
No	1463 (94.6)	1266 (95.1)	958 (90.6)	1155 (90.8)
Yes	83 (5.4)	65 (4.9)	99 (9.4)	117 (9.2)
*State/Territory of residence*				
New South Wales	652 (42.2)	564 (42.4)	526 (49.8)	614 (48.3)
Queensland	452 (29.2)	395 (29.7)	258 (24.4)	315 (24.8)
South Australia	196 (12.7)	164 (12.3)	119 (11.3)	151 (11.9)
Tasmania	56 (3.6)	47 (3.5)	29 (2.7)	38 (3.0)
Northern Territory	8 (0.5)	7 (0.5)	5 (0.5)	6 (0.5)
Australian Capital Territory	31 (2.0)	31 (2.3)	22 (2.1)	22 (1.7)
Western Australia	151 (9.8)	123 (9.2)	98 (9.3)	126 (9.9)
*Highest educational qualification*				
No schooling	–	–	–	–
Did not complete primary school	6 (0.4)	4 (0.3)	–	2 (0.2)
Completed primary school	24 (1.6)	19 (1.4)	10 (0.9)	15 (1.2)
Year 10 or equivalent	163 (10.5)	146 (11.0)	87 (8.2)	104 (8.2)
Year 11 or equivalent	41 (2.7)	33 (2.5)	19 (1.8)	27 (2.1)
Year 12 or equivalent	235 (15.2)	186 (14.0)	159 (15.0)	208 (16.4)
A trade, technical certificate or diploma	489 (31.6)	430 (32.3)	251 (23.7)	310 (24.4)
A university or college degree	421 (27.2)	375 (28.2)	372 (35.2)	418 (32.9)
Postgraduate qualifications	167 (10.8)	138 (10.4)	159 (15.0)	188 (14.8)
*Work status*				
Work full-time	585 (37.8)	496 (37.3)	566 (53.5)	655 (51.5)
Work part-time or casual	256 (16.6)	217 (16.3)	182 (17.2)	221 (17.4)
Full-time student	27 (1.7)	22 (1.7)	34 (3.2)	39 (3.1)
Unemployed and looking for work	69 (4.5)	64 (4.8)	61 (5.8)	66 (5.2)
Full-time home duties	102 (6.6)	89 (6.7)	44 (4.2)	57 (4.5)
Retired	447 (28.9)	392 (29.5)	132 (12.5)	187 (14.7)
Sick or on a disability pension	41 (2.7)	32 (2.4)	26 (2.5)	35 (2.8)
Other	19 (1.2)	19 (1.4)	12 (1.1)	12 (0.9)
*Occupation*				
Manager	287 (18.6)	240 (18.0)	252 (23.8)	299 (23.5)
Professional	375 (24.3)	327 (24.6)	259 (24.5)	307 (24.1)
Technician or trade worker	114 (7.4)	99 (7.4)	79 (7.5)	94 (7.4)
Community or personal service worker	90 (5.8)	79 (5.9)	69 (6.5)	80 (6.3)
Clerical or administrative worker	268 (17.3)	240 (18.0)	138 (13.1)	166 (13.1)
Sales worker	123 (8.0)	113 (8.5)	104 (9.8)	114 (9.0)
Machinery operator and driver	58 (3.8)	50 (3.8)	20 (1.9)	28 (2.2)
Labourer	139 (9.0)	106 (8.0)	101 (9.6)	134 (10.5)
Small business operator	92 (6.0)	77 (5.8)	35 (3.3)	50 (3.9)
*Marital status*				
Single or never married	327 (21.2)	269 (20.2)	298 (28.2)	356 (28.0)
Separated or divorced	135 (8.7)	128 (9.6)	74 (7.0)	81 (6.4)
Widowed	54 (3.5)	49 (3.7)	17 (1.6)	22 (1.7)
Married or living with partner (de facto)	1030 (66.6)	885 (66.5)	668 (63.2)	813 (63.9)
*Household composition*				
Single person	351 (22.7)	308 (23.1)	249 (23.6)	292 (23.0)
One parent family with children	77 (5)	71 (5.3)	74 (7.0)	80 (6.3)
Couple with children	520 (33.6)	426 (32.0)	408 (38.6)	502 (39.5)
Couple with no children	525 (34.0)	469 (35.2)	266 (25.2)	322 (25.3)
Group household (i.e. living with two or more people to whom you are NOT related)	73 (4.7)	57 (4.3)	60 (5.7)	76 (6.0)
*Annual personal income*				
$0 to $19,999	244 (15.8)	217 (16.3)	137 (13.0)	164 (12.9)
$20,000 to $39,999	402 (26.0)	348 (26.1)	200 (18.9)	254 (20.0)
$40,000 to $59,999	233 (15.1)	202 (15.2)	188 (17.8)	219 (17.2)
$60,000 to $79,999	238 (15.4)	192 (14.4)	161 (15.2)	207 (16.3)
$80,000 to $99,999	160 (10.3)	132 (9.9)	113 (10.7)	141 (11.1)
$100,000 to $119,999	102 (6.6)	97 (7.3)	99 (9.4)	104 (8.2)
$120,000 to $139,999	60 (3.9)	52 (3.9)	58 (5.5)	66 (5.2)
$140,000 to $159,999	39 (2.5)	38 (2.9)	46 (4.4)	47 (3.7)
$160,000 to $179,000	24 (1.6)	15 (1.1)	18 (1.7)	27 (2.1)
$180,000 or more	44 (2.8)	38 (2.9)	37 (3.5)	43 (3.4)
*Annual household income*				
$0 to $19,999	70 (4.5)	56 (4.2)	48 (4.5)	62 (4.9)
$20,000 to $39,999	287 (18.6)	255 (19.2)	145 (13.7)	177 (13.9)
$40,000 to $59,999	226 (14.6)	209 (15.7)	156 (14.8)	173 (13.6)
$60,000 to $79,999	214 (13.8)	166 (12.5)	137 (13.0)	185 (14.5)
$80,000 to $99,999	166 (10.7)	152 (11.4)	136 (12.9)	150 (11.8)
$100,000 to $119,999	149 (9.6)	125 (9.4)	133 (12.6)	157 (12.3)
$120,000 to $139,999	103 (6.7)	91 (6.8)	89 (8.4)	101 (7.9)
$140,000 to $159,999	127 (8.2)	102 (7.7)	84 (7.9)	109 (8.6)
$160,000 to $179,000	47 (3.0)	45 (3.4)	38 (3.6)	40 (3.1)
$180,000 or more	157 (10.2)	130 (9.8)	91 (8.6)	118 (9.3)
*Residence*				
Capital city and surrounds	1003 (64.9)	864 (64.9)	766 (72.5)	905 (71.1)
Regional town with more than 10,000 persons	396 (25.6)	341 (25.6)	223 (21.1)	278 (21.9)
A rural or remote location	147 (9.5)	126 (9.5)	68 (6.4)	89 (7.0)

Note: PGSI and SGHS are highly correlated indicators, treated in parallel in subsequent analyses

### Measures

All participants completed the following measures. Problem gambling status was assessed using the PGSI. The PGSI uses nine items (e.g. have you bet more than you could really afford to lose?) with each item measured on a four-point scale (from 0 = never to 3 = almost always). Total scores are summed and risk categories are yielded (non-problem 0, LR 1-2, MR 3-7, PG 8+) [2]. Reliability for the PGSI was high in the current sample (α = 0.95).

Gambling harm was assessed using the SGHS. The SGHS comprises 10-items (e.g. had regrets that made me feel sorry about my gambling) each measured in a binary no/yes format. The SGHS captures financial, emotional/psychological, and relationship harms due to gambling and yields scores 0-10 [3] however the screen does not specify categories. Nonetheless, recent research assessing the SGHS using the Personal Wellbeing Index suggests that cut-offs of 1-2, 3-5, 6+ provide a reasonable categorisation of differing degrees of harm [16]. Reliability for the SGHS was high in the current sample (α = 0.90).

We measured health utility using the SF-6D (see [32] for a detailed description). The SF-6D is a preference-based measure derived from the SF-12 item self-report measure [33]. It captures physical functioning, role limitations, social functioning, pain, mental health, and vitality, and yields health utility coefficients between 0.345 to 1.000 [34].

Demographic characteristics identified as risk factors for gambling problems and harms [17] were considered for inclusion in the propensity model: gender, country of birth, personal and parent’s highest level of education achieved, selected work status flags (FT student, unemployed, being unable to work due to infirmity, labourer), marital status, household composition (e.g. single, couple with children), personal and household income, and metropolitan/regional/rural residential location. Psychological risk factors such as cognitive style or rash impulsivity were measured but excluded due to potential endogeneity, particularly with respect to gambling problems when considered as a mental health condition.

The following key co-morbidities that affect health were also measured: excessive alcohol consumption (AUDIT-C) [35], any recreational drug use, cigarette smoking frequency (single-item measures), and ever having been diagnosed with a mood disorder, anxiety disorder, personality disorder, or any other mental health disorder (separate binary indicators). The AUDIT-C is a three-item measure of hazardous drinking (e.g. how often do you have six or more standard drinks on one occasion) with each item measured on a five-point scale. Reliability for the AUDIT-C in the current sample was (α = 0.67).

### Statistical analysis

The analyses took a multi-step approach, and all analyses were conducted for SGHS as well as PGSI. Because SGHS and PGSI are correlated, there is significant overlap between the affected and control groups for the two measures. The first step was to determine the required weights for the propensity score matching, which was based on initial logistic regressions predicting SGHS or PGSI (0 vs 1+; “Propensity” models in Table 2). Based on these regression results, propensity score weights were used in subsequent analyses predicting SF-6D scores using SGHS (and PGSI separately) as independent variables. Known risk factors were included as covariates (“Causal” models in Table 2). A final set of models was run predicting SF-6D using SGHS (and PGSI separately) as independent variables, but without the risk factors as covariates, to determine the effect of the covariates on the estimated decrements.

**Table 2:** Model summaries and beta coefficients for propensity and causal models of health utility scores

		Beta coefficients (SE)					
Model		Propensity		Causal		Causal (no covariates)	
DV		SGHS (0 vs 1+)	PGSI (0 vs 1+)	SF-6D	SF-6D	SF-6D	SF-6D
Regression type		Logistic	Logistic	OLS	OLS	OLS	OLS
DV		SGHS	PGSI	SGHS	PGSI	SGHS	PGSI
Constant		2.042*** (0.257)	2.435*** (0.235)	0.836*** (0.009)	0.832*** (0.009)	0.803*** (0.004)	0.804*** (0.004)
Gambling harms	(0) None (*n* = 1546)			–		–	
(SGHS)	(1-2) Low (*n* = 370)			−0.020** (0.006)		−0.022** (0.007)	
	(3-5) Moderate (*n* = 368)			−0.062*** (0.007)		−0.075*** (0.007)	
	(6-10) High (*n* = 319)			−0.109*** (0.007)		−0.153*** (0.008)	
Gambling problems	(0) Non-problem NP (*n* = 1331)				–		–
(PGSI)	(1,2) Low risk LR (*n* = 399)				−0.005 (0.006)		−0.007 (0.007)
	(3-7) Moderate risk MR (*n* = 438)				−0.051*** (0.006)		− 0.066*** (0.007)
	(8+) Problems PG (*n* = 435)				−0.099*** (0.007)		−0.137*** (0.007)
Alcohol consumption	Non-drinker			–	–		
(AUDIT-C)	(0-3) Non-risky			0.008* (0.007)	0.011* (0.008)		
	(4+) Risky			0.007 (0.007)	0.011 (0.007)		
Age (polynomial)	Linear (1)	−0.034*** (0.003)	−0.037*** (0.003)	0.278* (0.124)	0.111 (0.123)		
	Quadratic (2)			−0.251 (0.118)	−0.365** (0.116)		
	Cubic (3)			−0.235 (0.116)	−0.246* (0.113)		
Gender	Male	–	–	–	–		
	Female	−0.160 (0.107)	−0.251 (0.090)	− 0.016*** (0.005)	−0.019*** (0.005)		
Country of birth	Overseas	–	–	–	–		
	Australia	−0.164 (0.107)	−0.264* (0.105)	− 0.015* (0.006)	−0.008 (0.006)		
Education	Secondary or less	–	–				
	Trade/Cert	−0.091 (0.114)	−0.227* (0.111)				
	Tertiary	0.212 (0.120)	−0.110 (0.119)				
	Postgrad	0.344* (0.153)	0.200 (0.154)				
Unemployed (ref = no)			−0.284 (0.195)				
Personal income		0.111*** (0.029)	0.079** (0.029)				
Household income		−0.128*** (0.025)	−0.095*** (0.024)				
Mother’s highest education achieved		−0.063*** (0.019)					
Sick or on a disability pension (ref = no)				−0.126*** (0.015)	−0.133*** (0.015)		
Recreational drug use (ref = no)				−0.020* (0.008)	−0.015 (0.008)		
Cigarettes consumed per day	Non-smoker (0)			–	–		
	< 10			−0.014* (0.007)	−0.019** (0.007)		
	10+			−0.010 (0.007)	−0.018* (0.007)		
Past year diagnosis of … (ref = no)	Mood disorder			−0.049*** (0.009)	−0.053*** (0.009)		
	Anxiety disorder			−0.066*** (0.007)	−0.071*** (0.007)		
	Personality disorder			−0.017 (0.013)	−0.023 (0.013)		
	Any other psych. Disorder			−0.037** (0.012)	−0.027* (0.012)		
	Observations	2603	2603	2603	2603	2603	2603
	Adjusted R2			0.292	0.288	0.148	0.132
	Residual Std. Error			0.165	0.163	0.181	0.181
	Model df			18, 2584	18, 2584	3, 2599	32,599
	F			60.693***	59.427***	151.3***	132.9***

Note: Propensity models are unweighted. Case weights for the causal models (both with and without covariates) calculated from estimated probabilities from the propensity models. * *p* < 0.05, *p* < 0.01, *p* < 0.001

The working for the weights in the causal models is based on the binomial logistic regression predicting harm (SGHS = 1+) compared to not experiencing harm (SGHS = 0), or the equivalent for PGSI (0 vs 1+). The predictors in the models were known risk factors for experiencing gambling harm or problems and were chosen for each model based on backwards stepwise elimination using the Akaike Information Criteria, to avoid redundancy and multicollinearity. The models for SGHS and PGSI therefore had slightly different predictors to each other. While stepwise variable elimination has limitations for interpreting covariates, they do not apply in this case because our objective was not to interpret these covariate effects, but to achieve statistical control.

From the logistic regressions, predicted probability of harm (or problems) was derived for each individual and then cases in each group (affected vs control) were inversely weighted with respect to these group propensities based on the standard propensity weighting method:

$${\displaystyle \begin{array}{c}\mathrm{if}\left(\mathrm{affected}\right):\frac{1}{\hat{P}\left(\mathrm{affected}\right)}\\ {}\mathrm{if}\left(\mathrm{control}\right):\frac{1}{1-\hat{P}\left(\mathrm{affected}\right)}\end{array}}$$

This weighting acts to remove some potential selection bias from confounders in estimating the direct effect of gambling harm on health. This is because people with different demographic and other characteristics differ in their propensity to experiencing harm or problems from gambling, and these same risk factors can also contribute directly to lower wellbeing. For example, from Table 2, younger people in the present study were more likely to experience some degree of harm. The propensity weighting balances the groups of who were *actually* affected / unaffected, with respect to their *propensity* for being affected by gambling harms or problems. For example, looking at Table 1, affected gamblers were more likely to be younger compared to controls. The process “weights down” younger respondents in the affected group, and “weights up” younger respondents in the control group, balancing the groups with respect to this particular risk factor. One issue with propensity weighting is that excessively large or small weights can lead to outside case influence. However, skew and outliers of weights were moderate (median ~ 1.8, mean ~ 2, max ~ 6), so no thresholding of excessively large weights was required.

In a supplementary analysis, shown in Table 3, the empirically derived estimates were applied to population estimates of SGHS and PGSI score prevalence using a recent Victorian prevalence dataset [13], in order to estimate population aggregate impact. Finally, a standard Pearson correlation matrix (see Additional file 1), was calculated for descriptive purposes.

**Table 3:** Burden of harm estimates by PGSI and SGHS categories

	PGSI	Prevalence in Victorian gamblers	SF-6D utility weight (Current study, comorbidity controlled)	SF-6D utility weight [31]	Aggregate impact (DALYs)	Implied proportion of total population impact
PGSI	LR (1-2)	9.7%	−.005 ns	−.030	2874	14.6%
	MR (3-7)	3.5%	−.050*	−.057*	10,372	52.6%
	PG (8+)	1.1%	−.099*	−.181*	6454	32.8%
**TOTAL**		**14.3%**			**19,700**	**100%**
SGHS	Low (1-2)	7.1%	−.020*		8416	42.2%
	Moderate (3-5)	1.6%	−.061*		5784	29.0%
	High (6-10)	0.9%	−.108*		5761	28.8%
**TOTAL**		**9.6%**			**19,961**	**100%**

Note: Weights from Moayeri [31] provided for comparison only, and not used for subsequent calculations. SF-6D decrement (or disability) weights were sourced from Table 2 above, and control for other variables. Prevalence figures for the PGSI & SGHS in the Victorian community were sourced from Rockloff et al. [13], based on respondents who gambled in the last 12 months. Aggregate based on population of Victorian adults from census data: 5,926,624 x prevalence x SF-6D decrement, to form an estimate of per-year, disability adjusted life years (DALYs)

### Ethics

Ethical approval for this study was received from the institutional Human Research Ethics Committee (#22341) and all methods were performed in accordance with the relevant guidelines and regulations. Participants provided informed consent before participating.

## Results

The mean SF-6D health utility score for the entire sample was .769, which was similar to the mean of .763 from the 2009/2019 wave of the Household, Income and Labour Dynamics in Australia (HILDA) survey (*n* = 17,630) [36].

Table 2 provides model summaries for the propensity (columns 1-2) and causal (columns 3-4) components of SF-6D for the SGHS and the PGSI.

**Fig. 1:**
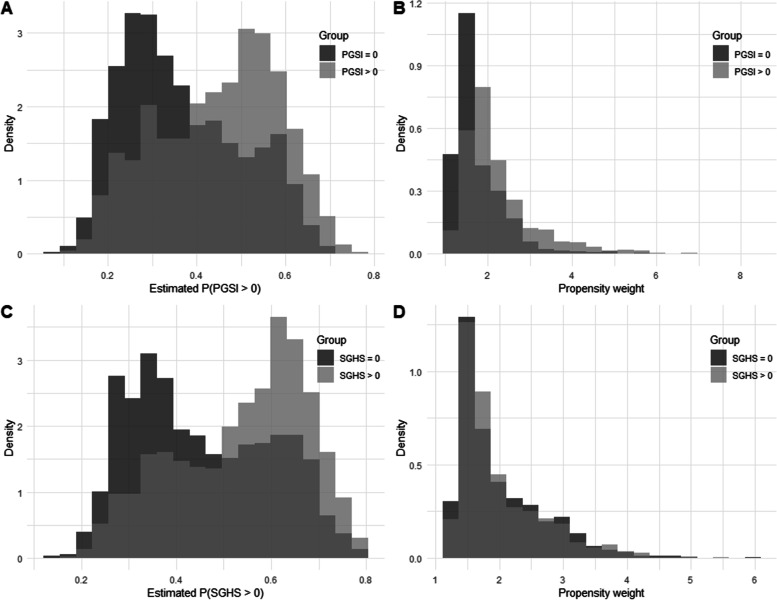
Distribution of the estimated probability of being in the affected group, for PGSI (**A**) and SGHS (**C**), and associated derived propensity weights used in the causal model (**B**, **D**). Note: The medium grey is simply overlap between the two distributions

For both screens, affected gamblers were significantly more likely to be younger, have a higher personal income, but lower household income. Those people scoring 1+ on the PGSI were less likely to have a trade/certificate level education than controls, while those scoring 1+ on the SGHS were more likely to have a postgraduate qualification than controls. Figure 1 (panels A, C) shows a histogram of the predicted probability of being in the affected group (versus controls) for the PGSI and SGHS respectively, and the corresponding weights (panels B, D). The most important control covariates were comorbidities: having an anxiety or mood disorder (approximate −.05 to −.07 decrement to health utility) and being sick or on a disability pension (approximately −.13 decrement).

SGHS and PGSI causal models both accounted for about 29% of variability in health utility. Causal effects for Low (1-2), moderate (3-5) and high (6-10) degrees of gambling harm (SGHS) were all significant, and estimated as −.020, −.062 and − .109, respectively. Causal effects for LR, MR and PGs (PGSI) were − .005, −.051 and − .099, respectively. Only the effects for MR and PG were significant decrements. To check for the influence of covariates, we re-ran the analyses without any covariates. We found the same pattern of significant / non-significant effects for SGHS and PGSI categories and only slightly larger magnitude decrement weights, with 13.2% of variance in SF-6D scores explained by the PGSI alone, and 14.8% explained by the SGHS.

Table 3 combines these per-person utility decrements with prevalence estimates from a recent population survey in Victoria, Australia to yield a basic calculation of population aggregate impact. It is important to note that because the prevalence of LR and low-harmed individuals is much higher than more severe categories, uncertainty associated with aggregate impact is more pronounced for this group.

## Discussion

This propensity score weighting study is the first to evaluate population gambling screens using health utility as the criterion outcome, propensity score matching and control for co-morbidities. For the PGSI, this yielded a similar pattern of decrements to that calculated by Moayeri [31] from the Household Income and Labour Dynamics in Australia dataset (Table 3). The smaller effect sizes found in the present study, especially for PG, appear to primarily be due to the additional measures of controlling for comorbid conditions. The use of an experienced utility framework complements prior elicitation of preference-based utility for the PGSI only [25, 27, 28], and contributes to literature validating the SGHS as an index of gambling-related harm. A key limitation is that these estimates are not directly comparable to preference-based utilities in terms of raw magnitudes, due to a variety of methodological differences. Nevertheless, the results are consistent in relative terms, in that per-person impacts to MR problematic gamblers, measured with the PGSI, and moderately-harmed gamblers, measured with the SGHS, are about half that of those in the most severe categories. However, while the decrement to those in the least severe SGHS group was statistically significant, no significant decrement to health-utility was found for LR gamblers identified by the PGSI. This may, of course, simply be a matter of relative power for the two scales.

Both the SGHS and the PGSI yielded consistent results in total population impact, and in finding that less than one-third (~ 6000 DALYS) of the population impact was attributable to the small proportion of gamblers identified as being in the most severe category of either instrument. Thus, the discrepancy between the two instruments is largely reflected in the attribution of utility decrements to the moderate and low categories. Further insight is gained by taking into account the differing sensitivity of the instruments, as indicated by the prevalence column in Table 3. Contrary to suggestions that the SGHS might ‘lower the bar’ for harm [5], the PGSI identifies *more* affected gamblers across all categories (14.3% 1+) than the SGHS (9.6% 1+), particularly in the LR and MR groups. Given the aggregate population impact is commensurate at about 19,800 DALYs, this indicates that the SGHS is a more specific instrument for identifying harmed individuals. On the other hand, the non-significant difference for LR gamblers supports a degree of scepticism regarding whether or not LR gamblers experience a meaningful degree of harm. The ability of the SGHS to identify statistically significant health decrements at low levels of harm is consistent with the theory underlying its development [3, 25], which was to specifically target harmful outcomes from excessive gambling, rather than the broader concepts of risky, uncontrolled, or problem gambling.

### Limitations and future directions

The study used experienced utility as the key outcome, propensity score matching of affected and unaffected individuals, and controlled for known comorbidities so as not to over-attribute associated SF-6D decrements to gambling. To the author’s knowledge, this is the best approach for estimating health utility impacts attributable to gambling problems or harms from cross-sectional self-report data. It provides a useful complement to the directly elicited preference-based utilities elicited for the PGSI in prior work [27]. It is also arguably more conservative than the results of Moayeri [31], avoiding both over-attribution and the stigma and framing effects involved in direct assessment of the impact of gambling. Nevertheless, the statistical techniques employed here are by no means a ‘silver bullet’ for achieving unbiased causal or counterfactual estimates from cross-sectional data [37].

The SF-6D, which is calculated from responses to the SF-12, has the advantage of yielding health utility scores on a genuine metric suitable for summation over individuals to create an index of population impact. However, it is arguably not perfectly suited to assessing the full scope of impacts to wellbeing and life-satisfaction caused by gambling. It includes items pertaining to physical pain and physical functioning, which we would not necessarily expect to be affected even by quite severe gambling problems. Thus, this can make it relatively insensitive compared to other benchmarks, such as measures of psychological distress or personal wellbeing. Future counterfactual studies might consider using a broader suite of outcomes that capture wellbeing, happiness and life-satisfaction. This would maintain the advantage of being an independent ‘yardstick’ for gambling-specific screens, at the expense of not necessarily yielding results on a metric scale.

Participants were drawn from a commercial panel provider, and opted into the study. Although demographic characteristics were reasonably typical of the Australian population, they did not comprise a random representative sample. As discussed elsewhere [38], virtually all sampling in the social sciences, including random digit dial computer-assisted telephone interviews, are not truly population representative. Nevertheless, the likely characteristics of those who are drawn to enrol in a commercial panel, such as having free time or requiring supplemental income, should be borne in mind when generalising to the population.

Finally, it is important to emphasise that gambling problems are known to lead to long-term financial, social and emotional impacts to the gambler and those around them. The present study was only designed to assess the ‘instantaneous’ health-related impact of the gambler who is currently reporting some degree of harm or problems. It does not measure economic impacts, legacy impacts, or harms to others.

In sum, understanding the impacts of gambling on health and wellbeing requires synthesising evidence from a variety of sources and methodologies. The current study provides a new reference point in this ongoing effort, but should not be taken as overriding or replacing knowledge gained from prior quantitative or qualitative approaches.

## Conclusion

Hitherto, the PGSI and the SGHS have provided only qualitative categorisation of affected gamblers, leading to dispute as to how non-zero scores on these instruments should be interpreted in terms of *how much* impact we should infer, given these scores. Consequently, prevalence surveys of gambling problems and harm have been limited to describing the prevalence of gamblers across nominal categories without strong guidance for a meaningful interpretation of these categories. Prior work employing direct elicitation of health impact has been criticised for being vulnerable to various forms of biases. This paper presents indirect estimates of experienced health utility decrements attributable to gambling, a method that overcomes these limitations. All non-zero increasing scores on the SGHS are associated with progressively larger decrements to health. Those reporting a high degree of harm on the SGHS (6+) experience around 5 times the impact as those in the low range (1-2). However, this impact varies inversely with the prevalence of individuals in these categories, yielding a similar ‘burden’ of population impact across the spectrum of harm. Broadly similar results were found for the PGSI, with the important exception that no significant decrement was detected for LR gamblers, and relatively greater burden attributable to MR gamblers. Since the decrement associated with PGs is almost exactly double that of MR gamblers, a reasonable heuristic when using the PGSI is to weight these two categories accordingly in statistical calculations. The SGHS yields a similar population-aggregate estimate of the ‘burden of gambling harm’ compared to the PGSI (~ 20,000 DALYs per annum in Victoria Australia), but confines this impact to 9.6%, rather than 14.3% of gamblers. For these individuals, there is a spectrum of harm, with progressively fewer individuals experiencing a greater degree of impact. The methodological choices of this paper, including the use of the SF-6D benchmark, reliance on experienced-utility, and the propensity weighting methodology were all geared towards a conservative estimate of impact from gambling. Further work applying this framework could consider a broader range of outcomes, consider life-course and legacy impacts, and – perhaps most critically – also consider harm to others. Importantly, this study shows that the SGHS and the PGSI have broadly similar performance in identifying decrements to health-utility from engagement with gambling, and demonstrates that the SGHS does not overestimate harm in the community relative to the PGSI.

Especially given the purported commitment of gambling research to a public health model, it is somewhat remarkable that this is one of the first studies to attempt to link standard gambling screens of problems and harms to established measure of impact to health and wellbeing. Most government policies are geared towards reducing negative impacts of gambling, i.e., maximising health utility in the affected communities. Therefore in our view, the success (or failure) of policy is properly evaluated by monitoring aggregate changes in health utility. Population-weighted scoring of the PGSI or the SGHS for this purpose may be done by applying the health utility decrements estimated here. Gambling research has also suffered from the terminology of ‘low / moderate risk’ (of gambling problems) applied to intermediate PGSI categories, which implies that they are not currently experiencing negative impact (see [39] for a detailed discussion of this issue). At least in the case of the moderate risk category, these individuals suffer a detectable degree of harm; and in aggregate, contribute more to population impact than the more severe but less prevalent ‘problem gambler’ cohort. The present study did not preferentially sample those in the low risk category, and the relatively small per-person impact was not statistically detectable. However, given their relatively high prevalence in the population this does not imply the aggregate impact to this group is zero or negligible. Further work to specifically study this group is warranted.

## Supplementary Information

**Additional file 1.**

## Data Availability

The datasets generated and/or analysed during the current study are not publicly available as this would breach a condition of the ethical approval obtained, but are available from the corresponding author (Matthew Browne m.browne@cqu.edu.au) on reasonable request.

## Abbreviations

AUDIT-C: Alcohol Use Disorders Identification Test - Consumption
DALY: Disability Adjusted Life Year
HILDA: Household, Income and Labour Dynamics in Australia
LR: Low-risk
MR: Moderate-risk
NPG: Non-problem gambler
PG: Problem gambler
PGSI: Problem Gambling Severity Index
PWI: Personal Wellbeing Index
SF-6D: Short Form Six-Dimension
SGHS: Short Gambling Harms Screen

## Footnotes

1 Gambling included participating in at least one of the follow activities within the past 12 months: race betting, electronic gaming machines (pokies), casino table games, sports betting, informal private betting for money, Keno, bingo, esports betting, and fantasy sports betting.
