# Supplementary material for: Benchmarking gambling screens to health-state utility: the PGSI and the SGHS estimate similar levels of population gambling-harm
Source: BMC Public Health. 2022 Apr 27;22:839. doi: 10.1186/s12889-022-13243-4 (PMC9044680; doi:10.1186/s12889-022-13243-4)
Supplement: Supplementary file 1 — Additional file 1. [file 12889_2022_13243_MOESM1_ESM.docx]

**Additional material**

- File name: Additional file 1. File format: .docx. Title of data: Correlation matrix. Description of data: Standard Pearson correlations for descriptive purposes

|  | **Outcome** |  | **Indicators** | |  | | | **Covariates** | | | | | | | | | | | | | | | |  | | **Comorbidities** | | | | | |  |
| --- | --- | --- | --- | --- | --- | --- | --- | --- | --- | --- | --- | --- | --- | --- | --- | --- | --- | --- | --- | --- | --- | --- | --- | --- | --- | --- | --- | --- | --- | --- | --- | --- |
| **Variables** | (1) |  | (2) | (3) | |  | (4) | (5) | (6) | (7) | (8) | (9) | (10) | (11) | (12) | (13) | (14) | (15) | (16) | (17) | (18) |  | (19) | | (20) | | (21) | (22) | (23) | (24) | (25) | |
| (1) SF-6D score | 1.00 |  |  |  | |  |  |  |  |  |  |  |  |  |  |  |  |  |  |  |  |  |  | |  | |  |  |  |  |  | |
|  |  |  |  |  | |  |  |  |  |  |  |  |  |  |  |  |  |  |  |  |  |  |  | |  | |  |  |  |  |  | |
| (2) SGHS score | -0.39 |  | 1.00 |  | |  |  |  |  |  |  |  |  |  |  |  |  |  |  |  |  |  |  | |  | |  |  |  |  |  | |
| (3) log(PGSI) | -0.39 |  | 0.79 | 1.00 | |  |  |  |  |  |  |  |  |  |  |  |  |  |  |  |  |  |  | |  | |  |  |  |  |  | |
|  |  |  |  |  | |  |  |  |  |  |  |  |  |  |  |  |  |  |  |  |  |  |  | |  | |  |  |  |  |  | |
| (4) Male | 0.10 |  | 0.01 | 0.02 | |  | 1.00 |  |  |  |  |  |  |  |  |  |  |  |  |  |  |  |  | |  | |  |  |  |  |  | |
| (5) Australian born | -0.07 |  | 0.01 | 0.02 | |  | 0.02 | 1.00 |  |  |  |  |  |  |  |  |  |  |  |  |  |  |  | |  | |  |  |  |  |  | |
| (6) Age | 0.18 |  | -0.23 | -0.31 | |  | 0.13 | -0.13 | 1.00 |  |  |  |  |  |  |  |  |  |  |  |  |  |  | |  | |  |  |  |  |  | |
| (7) Education | -0.02 |  | 0.11 | 0.13 | |  | 0.09 | -0.07 | -0.22 | 1.00 |  |  |  |  |  |  |  |  |  |  |  |  |  | |  | |  |  |  |  |  | |
| (8) Full time student | -0.06 |  | 0.03 | 0.04 | |  | -0.06 | 0.00 | -0.20 | -0.04 | 1.00 |  |  |  |  |  |  |  |  |  |  |  |  | |  | |  |  |  |  |  | |
| (9) Unemployed | -0.07 |  | 0.07 | 0.03 | |  | -0.03 | -0.01 | -0.10 | -0.11 | -0.04 | 1.00 |  |  |  |  |  |  |  |  |  |  |  | |  | |  |  |  |  |  | |
| (10) Sick or on disability pension | -0.20 |  | 0.01 | 0.00 | |  | 0.00 | 0.02 | 0.05 | -0.11 | -0.03 | -0.04 | 1.00 |  |  |  |  |  |  |  |  |  |  | |  | |  |  |  |  |  | |
| (11) Labourer | 0.00 |  | 0.02 | 0.03 | |  | 0.04 | 0.02 | -0.05 | -0.22 | 0.04 | 0.15 | 0.02 | 1.00 |  |  |  |  |  |  |  |  |  | |  | |  |  |  |  |  | |
| (12) Married or living with partner (de facto) | 0.11 |  | -0.05 | -0.04 | |  | 0.01 | -0.07 | 0.15 | 0.09 | -0.16 | -0.15 | -0.07 | -0.08 | 1.00 |  |  |  |  |  |  |  |  | |  | |  |  |  |  |  | |
| (13) Household - Couple with children | -0.03 |  | 0.07 | 0.10 | |  | -0.02 | 0.02 | -0.20 | 0.15 | -0.06 | -0.07 | -0.03 | -0.02 | 0.46 | 1.00 |  |  |  |  |  |  |  | |  | |  |  |  |  |  | |
| (14) Personal income | 0.02 |  | 0.11 | 0.15 | |  | 0.21 | 0.07 | -0.25 | 0.44 | -0.10 | -0.18 | -0.14 | -0.17 | 0.16 | 0.23 | 1.00 |  |  |  |  |  |  | |  | |  |  |  |  |  | |
| (15) Household income | 0.05 |  | 0.04 | 0.07 | |  | 0.09 | 0.06 | -0.30 | 0.38 | -0.05 | -0.17 | -0.15 | -0.18 | 0.28 | 0.33 | 0.74 | 1.00 |  |  |  |  |  | |  | |  |  |  |  |  | |
| (16) Living in capital city / surrounds | 0.02 |  | 0.08 | 0.09 | |  | 0.04 | -0.07 | -0.19 | 0.22 | 0.02 | -0.02 | -0.09 | -0.05 | 0.00 | 0.08 | 0.18 | 0.20 | 1.00 |  |  |  |  | |  | |  |  |  |  |  | |
| (17) Father’s highest education achieved | -0.03 |  | 0.02 | 0.05 | |  | 0.05 | -0.03 | -0.15 | 0.24 | 0.05 | -0.01 | -0.05 | -0.06 | 0.01 | 0.06 | 0.17 | 0.13 | 0.10 | 1.00 |  |  |  | |  | |  |  |  |  |  | |
| (18) Mother’s highest education achieved | -0.04 |  | 0.01 | 0.06 | |  | 0.06 | -0.04 | -0.18 | 0.14 | 0.03 | -0.01 | 0.00 | -0.03 | -0.02 | 0.04 | 0.16 | 0.11 | 0.04 | 0.56 | 1.00 |  |  | |  | |  |  |  |  |  | |
|  |  |  |  |  | |  |  |  |  |  |  |  |  |  |  |  |  |  |  |  |  |  |  | |  | |  |  |  |  |  | |
| (19) AUDIT – Risky category | -0.10 |  | 0.09 | 0.10 | |  | 0.18 | 0.08 | -0.06 | 0.02 | -0.02 | -0.02 | -0.01 | -0.02 | 0.01 | 0.03 | 0.13 | 0.09 | 0.02 | 0.05 | 0.06 |  | 1.00 | |  | |  |  |  |  |  | |
| (20) Recreational drug use | -0.19 |  | 0.16 | 0.16 | |  | -0.04 | 0.07 | -0.20 | -0.01 | 0.03 | 0.09 | 0.04 | 0.05 | -0.05 | 0.03 | 0.03 | 0.02 | 0.01 | 0.06 | 0.07 |  | 0.17 | | 1.00 | |  |  |  |  |  | |
| (21) 10+ cigarettes per day | -0.13 |  | 0.12 | 0.11 | |  | 0.03 | 0.06 | -0.06 | -0.03 | -0.04 | 0.03 | 0.08 | 0.07 | -0.06 | 0.02 | 0.06 | 0.00 | -0.02 | 0.03 | 0.05 |  | 0.15 | | 0.21 | | 1.00 |  |  |  |  | |
| (22) Diagnosed mood disorder | -0.35 |  | 0.26 | 0.24 | |  | -0.06 | 0.07 | -0.13 | 0.04 | 0.06 | 0.03 | 0.10 | 0.01 | -0.06 | 0.03 | 0.03 | -0.02 | -0.05 | 0.02 | 0.04 |  | 0.09 | | 0.24 | | 0.13 | 1.00 |  |  |  | |
| (23) Diagnosed anxiety disorder | -0.38 |  | 0.23 | 0.21 | |  | -0.14 | 0.06 | -0.16 | -0.04 | 0.05 | 0.04 | 0.13 | -0.01 | -0.07 | 0.02 | -0.04 | -0.04 | -0.05 | 0.01 | 0.04 |  | 0.06 | | 0.24 | | 0.11 | 0.50 | 1.00 |  |  | |
| (24) Diagnosed personality disorder | -0.27 |  | 0.29 | 0.27 | |  | 0.02 | 0.07 | -0.12 | 0.08 | 0.06 | 0.00 | 0.08 | 0.01 | -0.04 | 0.05 | 0.07 | 0.01 | 0.02 | 0.03 | 0.04 |  | 0.11 | | 0.26 | | 0.16 | 0.43 | 0.30 | 1.00 |  | |
| (25) Diagnosed other psych disorder | -0.29 |  | 0.28 | 0.28 | |  | -0.01 | 0.08 | -0.15 | 0.07 | 0.06 | -0.01 | 0.10 | 0.01 | 0.00 | 0.08 | 0.06 | 0.02 | -0.01 | 0.03 | 0.04 |  | 0.09 | | 0.19 | | 0.12 | 0.42 | 0.35 | 0.49 | 1.00 | |
